# The Use of Technology Among Persons With Memory Concerns and Their Caregivers in the United States During the COVID-19 Pandemic: Qualitative Study

**DOI:** 10.2196/31552

**Published:** 2022-03-17

**Authors:** Elizabeth A Albers, Jude Mikal, Ashley Millenbah, Jessica Finlay, Eric Jutkowitz, Lauren Mitchell, Brenna Horn, Joseph E Gaugler

**Affiliations:** 1 Division of Health Policy and Management School of Public Health University of Minnesota Minneapolis, MN United States; 2 Social Environment and Health Program Institute for Social Research University of Michigan Ann Arbor, MI United States; 3 Department of Health Services, Policy and Practice School of Public Health Brown University Providence, RI United States; 4 Department of Psychology Emmanuel College Boston, MA United States

**Keywords:** social isolation, dementia, caregiving - informal, aging in place, caregivers, aging, elderly, pandemic, COVID-19, mental health, technology use, health technology

## Abstract

**Background:**

Stay-at-home orders and other public health measures designed to mitigate the spread of COVID-19 have increased isolation among persons with memory concerns (PWMCs: individuals diagnosed with cognitive impairment or Alzheimer disease or related dementias). The pandemic has also exacerbated challenges for family members who care for PWMCs. Although technology has demonstrated the potential to improve the social connections and mental health of PWMCs and their family caregivers (CGs), previous research shows that older adults may be reluctant to adopt new technologies.

**Objective:**

We aimed to understand why and how some PWMCs and their CGs altered their use of mainstream technology, such as smartphones and fitness trackers, and assistive technology to adapt to lifestyle changes (eg, increased isolation) during the COVID-19 pandemic.

**Methods:**

Using data collected in 20 qualitative interviews from June to August 2020 with 20 PWMCs and family CG dyads, we assessed changes in and barriers to everyday technology use following the implementation of COVID-19 mitigation strategies in the United States. Zoom videoconferencing was utilized to conduct the interviews to protect the health of the participants who were primarily older adults.

**Results:**

Using qualitative thematic analysis, we identified 3 themes that explained motivations for using technology during a pandemic: (1) maintaining social connections, (2) alleviating boredom, and (3) increasing CG respite. Results further revealed lingering barriers to PWMC and CG adoption of technologies, including: (1) PWMC dependence upon CGs, (2) low technological literacy, and (3) limitations of existing technology.

**Conclusions:**

This in-depth investigation suggests that technology can provide PWMCs with more independence and offer CGs relief from CG burden during periods of prolonged isolation.

## Introduction

Persons with memory concerns (PWMCs: individuals formally diagnosed with mild-to-moderate cognitive impairment or Alzheimer disease [AD] or Alzheimer disease–related dementias [ADRD]) and their family members who care for them experience significant challenges in their daily lives. Before the COVID-19 pandemic, PWMCs were likely to experience social isolation as changes in memory, social roles, and personality occurred [1]. Family caregivers (CGs) of PWMCs also had an increased risk of social isolation before the pandemic [2].

COVID-19 is a respiratory illness spread mainly through respiratory droplets and direct contact that is more likely to result in severe illness or death for older adults. Due to the unknown nature of COVID-19 at the time and rapid global spread, many services such as in-home aides and adult day services quickly closed at the onset of the pandemic [3]. This sudden absence of or disruption to home- and community-based services shifted the burden of continuous and comprehensive care to family CGs and intensified their existing challenges [4-6]. Since the onset of the pandemic, research has shown that CGs of PWMCs have experienced increased stress related to the exacerbation of PWMCs’ behavioral, psychological, or dementia symptoms [5]. The increased burden and stress family CGs experienced during the initial stages of the pandemic were related to the amount of social support they received, the level of help the PWMCs required to complete activities of daily living, and the level of CGs’ concern about the pandemic [7,8]. Throughout the course of the pandemic, many CGs have indicated concerns about a rapid decline in the cognitive functioning of PWMCs, due, in part, to the lack of social interaction [9].

Various types of technology have been used in research with PWMCs and family CGs. Assistive technology, designed specifically to assist PWMCs and their CGs perform a task, is associated with improved cognitive abilities and increased autonomy among PWMCs [10]. Mainstream technologies, such as Zoom or fitness trackers, also offer benefits to this population, such as by supporting social and physical functioning [11]. The use of technology to communicate with friends and family members allows for social connection while social distancing. Remote socialization, such as through web-supported Zoom videoconferencing, is associated with increased positive emotions and decreased agitation among PWMCs [10]. Information and communication technology use is positively associated with social connection and social support, as well as reduced social isolation among older adults [12].

In a prior study, 71% of CGs expressed interest in technology to support caregiving tasks [13]. However, interest in any given technology does not guarantee adoption. A technology’s perceived value and perceived impact on quality of life, an individual’s confidence in their ability to learn the technology, and social network support of technology use are key hurdles that influence technology adoption in this population [14,15]. Additionally, adoption of technologies for PWMCs and their CGs tends to be low due to barriers such as cost, complexity, inflexibility, a lack of awareness, and even age, income, and education [11,16,17].

During the COVID-19 pandemic, particularly during stay-at-home orders, the use of technology was necessary to sustain social connection and physical and mental health. Public health measures, such as social distancing, may have altered the perceived value of certain technologies on quality of life among many community-dwelling PWMCs and CGs. For example, telehealth medical visits were deemed feasible and acceptable to PWMCs and their CGs during the pandemic [18]. Social network support may have further influenced the uptake of certain technologies during the pandemic to maintain social connections. Therefore, previously identified barriers to technology adoption could have been outweighed by the increased social isolation and desire to maintain health among some PWMCs and their CGs.

The aim of this study was to investigate how and why some PWMCs and their CGs living in the community changed their mainstream and assistive technology use during the COVID-19 pandemic in the United States. Additionally, we aimed to understand how some PWMCs and their CGs used technology to adapt to isolation during the pandemic.

## Methods

### Recruitment

In total, 20 PWMCs and their CGs who lived in the community were recruited in the United States to assess (1) how their technology use shifted in response to the COVID-19 pandemic and (2) the impact that shift had on social isolation. Each PWMC-CG dyad was recruited to participate in a semistructured qualitative interview over Zoom videoconferencing. Although Zoom interviews were utilized to protect the health of older adult participants who were more likely to develop severe illness if they contracted COVID-19, this may have introduced selection bias by only including more technologically literate participants who could use Zoom [3]. Selection bias is discussed further in the Discussion section. Participants were recruited through the University of Minnesota Caregiver Registry, a list of family members of PWMCs and health professionals who have agreed to learn about research participation opportunities available on behalf of the senior author’s project team. We emailed all individuals in the registry, inviting them to participate in the study. Participants were also recruited through email advertisements in professional networks and at memory clubs and adult day programs for PWMCs.

**Figure 1:**
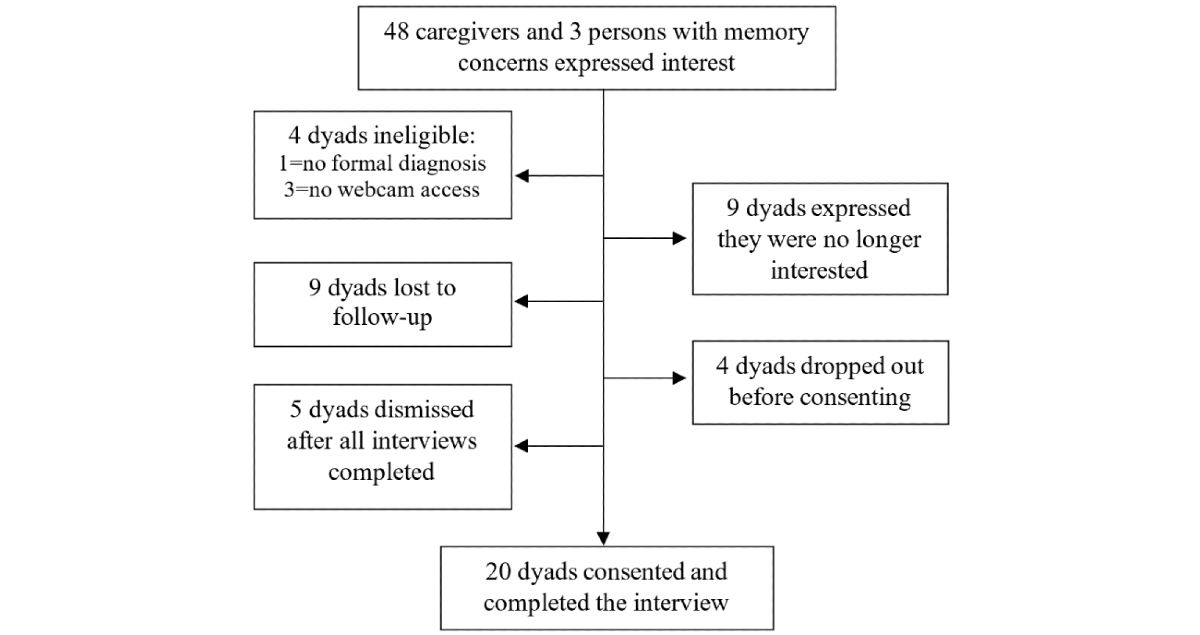
Study participant flowchart.

To be eligible to participate, the PWMCs had to speak English, have no history of a serious mental illness (ie, any major psychiatric disorder), and have a diagnosis of AD/ADRD or mild-to-moderate cognitive impairment by a physician. To be eligible, CGs had to speak English, be 21 years of age and over, and self-identify as someone who assists the PWMCs because of their memory loss. Because the interviews were conducted via Zoom, 3 dyads were considered ineligible since neither member of the dyad had access to a working web camera and microphone. One dyad was ineligible because the PWMC had never received a formal diagnosis of AD/ADRD or cognitive impairment. Figure 1 describes study participant flow. In total, 40 participants were enrolled and participated in the dyadic interviews conducted by authors EA and AM. The study was approved by the University of Minnesota Institutional Review Board (STUDY00006318).

### Data Collection Procedures

Qualitative interviews were conducted from June 25 to August 6, 2020. Before an interview began, the CGs and PWMCs had to provide verbal consent or assent. PWMCs’ capacity to consent was evaluated by administering the Mini-Cog and the University of California, San Diego (UCSD) Brief Assessment of Capacity to Consent (UBACC) [19,20]. The PWMCs provided verbal consent if they had a Mini-Cog score of 3 or higher and a UBACC score of 14.5 or higher. PWMCs provided verbal assent if they scored 2 or lower on the Mini-Cog or less than 14.5 on the UBACC. Once consent or assent was obtained, we administered a brief survey to CGs and PWMCs to determine age, sex, race, ethnicity, education, income, employment status, living arrangement, relationship to each other, and disease progression of the PWMCs.

Qualitative interviews adhered to a semistructured protocol focusing on 4 major topics: (1) technology use pre-COVID-19, (2) technology use during the COVID-19 pandemic, (3) adoption of specific technologies during the pandemic, and (4) facilitators and barriers to technology adoption. See Multimedia Appendix 1 for the semistructured interview questions. Interviews were audio-recorded, and direct observation notes were completed within 24 hours of each interview to document impressions of the participants’ location, level of comfort with Zoom technology, any nonverbal behaviors of importance, and how the PWMCs and CGs interacted.

### Data Analysis

Audio recordings were professionally transcribed and organized in NVivo 12 (QSR International). Interview transcripts and direct observation notes were thematically analyzed using Braun and Clarke’s 6 steps of thematic analysis: (1) familiarization, (2) generation of initial codes, (3) search for themes, (4) review themes, (5) define and name themes, and (6) write-up of themes analyzed [21]. An iterative process was followed to continually identify themes, linkages, and explanations, which were compared to create a codebook. Researchers then identified textual elements that emerged repeatedly (ie, codes); these codes were clustered into larger categories that were used to construct major thematic elements from the text. All authors reviewed the codebook to refine and clarify codes and themes. Next, EA and AM independently coded a subset of the interviews and revised the codebook after comparing codes and discussion with the full authorship team. EA coded the interview transcripts, and author JM reviewed the coded material and revised it, as necessary, to ensure accuracy and replicability. The iterative process of developing codes and themes ensured that saturation was achieved and the data were characterized appropriately. Peer debriefing, negative case analysis, and clear audit trails enhanced transparency and rigor in the analysis [22].

## Results

### Participants

In total, 20 dyads participated in the study (Table 1). Most PWMCs were male (14/20, 70%), and most CGs were female (14/20, 70%). In addition, 16 (80%) of the 20 dyads were spouses/partners, while the other dyads were siblings or parents/adult children. Furthermore, 18 (90%) of the 20 PWMCs lived with their CGs. Half (n=10) of the PWMCs were diagnosed with AD or early-onset AD, 6 (30%) were diagnosed with mild cognitive impairment, and the remaining 4 (20%) were diagnosed with other types of memory loss.

**Table 1:** Demographics of participants in the study.

Characteristics		Entire sample (N=40)	PWMCs^a^ (N=20)	CGs^b^ (N=20)
Age (years), mean (range)		72.23 (37-88)	74.75 (57-88)	69.70 (37-86)
**Race, n (%)**				
	White	38 (95.0)	19 (95.0)	19 (95.0)
	Black/African American	2 (5.0)	1 (5.0)	1 (5.0)
**Ethnicity, n (%)**				
	Hispanic	1 (2.5)	0	1 (5.0)
	Non-Hispanic	39 (97.5)	20 (100.0)	19 (95.0)
**Gender, n (%)**				
	Female	20 (50.0)	6 (30.0)	14 (70.0)
	Male	20 (50.0)	14 (70.0)	6 (30.0)
**Employment status, n (%)**				
	Employed	4 (10.0)	N/A^c^	4 (20.0)
	Retired	15 (37.5)	N/A	15 (75.0)
	Homemaker	1 (2.5)	N/A	1 (5.0)
**Education, n (%)**				
	High school degree	5 (12.5)	4 (20.0)	1 (5.0)
	Some college	12 (30.0)	5 (25.0)	7 (35.0)
	Bachelor’s degree or more	23 (57.5)	11 (55.0)	12 (60.0)

^a^PWMC: person with memory concerns.

^b^CG: caregiver.

^c^N/A: not applicable.

### Qualitative Analysis

Overall, 6 themes were identified that highlight why and how CGs and PWMCs altered their technology use during the COVID-19 pandemic. We present them in 3 groups: (1) facilitators of technology use, (2) barriers to technology use, and (3) overcoming challenges. Technological devices commonly used by participants were smartphones, smartwatches, computers, tablets, smart TVs, and assistive technology designed for PWMCs, such as pill dispensers.

#### Facilitators of Technology Use During the COVID-19 Pandemic

Due to stay-at-home orders and other public health measures to reduce the spread of COVID-19, PWMCs and CGs experienced physical and social isolation. Engagement with technology alleviated some of the negative outcomes of isolation by (1) sustaining social connections, (2) reducing boredom, and (3) increasing CG respite. These themes are presented with quotes and associated pseudonyms in Table 2 and discussed in more detail later.

**Table 2:** Facilitators of technology use during the COVID-19 pandemic for PWMCs^a^ and their CGs^b^.

Theme	Description	Exemplary quotes
Sustaining social connections	CGs and PWMCs were motivated to use technology to maintain social connections that would have otherwise been diminished during the pandemic.	“It's a necessity if we want to keep in touch with people. We can't go visiting family all the time. So, it makes us feel good to be able to see them and, like with you, have a conversation. We're not so isolated.” [Lisa, F^c^, 77 years, CG] “They’re about 6 of us that’ll text back and forth to one another a few times during the week.” [Tristan, M^d^, 71 years, PWMC] “We have a night set aside weekly that we all just kind of check-in with each other, just a videoconference.” [Hazel, F, 75 years, CG]
Reducing boredom	CGs and PWMCs used online games and streaming services and browsed the internet to promote entertainment and engagement.	“I got put on a game that I was playing constantly. That’s what got me by.” [Peyton, F, 37 years, CG] “We really have been very, very isolated, so when there's nothing to do, you spend more time on ways to reach out to people or just to get information.” [Rick, M, 73 years, CG] “For the first time a few months ago, we subscribed to Netflix and we’re taking advantage of that . . . there’s no more going to theaters.” [Darius, M, 82 years, CG]
Increasing CG respite	CGs used technology to alleviate or reduce caregiving tasks to potentially create more time for respite.	“When you have . . . 3 or 4 appointments on certain weeks, and then other weeks, there are 12 appointments. So, without technology [Slack], you can’t have your job and coordinate all these things.” [Louis, M, 47 years, CG] “I signed up for the GPS^e^ [watch] thing. That's worth a million dollars, you know . . . if he wants to go out by himself and I can track him” [Judy, F, 62 years, CG] “I am using the computer more for food purchases, but we still do go out, and then again, I’m using the watch to track where he’s at.” [Lily, F, 64 years, CG]

^a^PWMC: person with memory concerns.

^b^CG: caregiver.

^c^F: female.

^d^M: male.

^e^GPS: Global Positioning System.

#### Sustaining Social Connections

Almost every participant expressed how the pandemic changed how they connected with friends and family. Most of the participants started using Zoom or other videoconferencing technology during the pandemic, while some reported using videoconferencing before the pandemic to keep in touch with family or friends. Many participants indicated new or increasingly frequent engagement with others through computer-mediated communication (CMC), with some even communicating with family members with whom they had lost contact. Half of the dyads reported increased online social engagement, which helped maintain social connection and reduced negative effects of prolonged isolation. April (female [F], 62 years, CG) shared how she used videoconferencing to maintain some aspects of her social connections and said,

My mental health would definitely suffer without the technology of the Zoom meetings and videoconferencing capabilities.

Both PWMCs and CGs realized the importance of social connections during isolation and the importance of sustaining social connections through CMC.

Participants emphasized that ensuring the PWMCs remained socially connected through CMC use was important for mental health and to possibly slow dementia progression. Louis (male [M], 47 years, CG) shared his concern for his father’s lack of social connection during the pandemic:

When you have the connections, then you remember people’s names or who they are. If you don’t see people for a long time, then you start to forget who they are.

For those with the ability and resources to use CMC, it was instrumental in allowing PWMCs to maintain social connections during the pandemic.

Participants also mentioned an increase in the use of other technologies to stay connected, such as photo-sharing apps (eg, Snapchat), texting, and emailing. Barb (F, 86 years, CG) noted she does “a little more texting . . . and more email, because now they're sending all this stuff of what you can do while you're at home, instead of going to adult day [programs].” CGs and PWMCs demonstrated myriad ways in which they adapted to the isolation using different technologies to maintain social connections.

#### Reducing Boredom

Using technology to reduce boredom was common among participants. Some engaged more passively with technology through streaming movies and music. Some participants were actively engaged with technology by attending online classes, playing memory games, exercising to workout videos, or searching for medical facts. Layla (F, 72 years, PWMC) expressed gratitude for the numerous entertainment options available:

If we were going through this pandemic in the 1940s, it'd be an absolute mess, and [now] you can watch TV and see what's going on and all that stuff . . . it's a hard time, but in a sense there's still things we can do.

Reducing boredom was important for isolated CGs and PWMCs staying at home, which led to the increased use of existing technology and the adoption of new ones, along with continued cognitive engagement.

#### Increasing Caregiver Respite

Some CGs and PWMCs adopted new technologies that offered convenience while in the home, such as telehealth visits, grocery delivery services, and Global Positioning System (GPS) technology. These technologies reduced stress and saved time for CGs. Peggie (F, 60 years, CG) leveraged technology for telehealth visits and said,

I actually kind of like if we don’t really need to be in the office for a visit. It takes a lot less time . . . versus driving back and forth and waiting.

Telehealth visits offered CGs conveniences, as did online shopping. Lisa (F, 77 years, CG) began ordering groceries online because

Before [the pandemic], I had freedom for, like, a 4-hour slice twice a week. Eight hours where I could get out and do errands that I needed to do, and now I don't have that, and I don't like to leave Myles alone for any length of time. So, I just order my groceries online, and then I go pick them up or my daughter will pick them up for us.

Online shopping allowed CGs who usually shopped in person to not worry about leaving their relative in the car or at home alone for an extended period.

The loss of adult day programs and in-home care visits made finding personal time difficult for some CGs. In some cases, GPS technology provided CGs respite, while still allowing them to attend to the health and safety of the PWMC when needed. CGs, like Rick (M, 73 years) shared how he used GPS watch technology to track his wife who exercised outside without him being present. Rick said,

If Layla was walking and she didn't come back when I thought she should, and I had no clue where to look for her, that would be incredibly stressful!

The GPS watch provided Rick and his wife free time and a sense of independence. Another CG, Judy (F, 62 years) explained that when her relative wore a GPS watch it gave her “peace of mind.” Christa (F, 63 years, CG) shared that she used tracking technology because her relative lived in a different city and therefore was not able to know where her relative always was without the help of GPS. Through the adoption of technology, some CGs were able to find ways to create respite time for themselves.

#### Barriers to Technology Use During the COVID-19 Pandemic

Managing the COVID-19 pandemic in conjunction with caring for someone with memory concerns presented its own set of challenges, as did using new technology. Barriers that impacted CG and PWMC technology use included (1) limitations of existing technology, (2) technological literacy, and (3) the dependence on CGs to use technology. These themes are presented with quotes and associated pseudonyms in Table 3 and discussed in more detail later.

**Table 3:** Barriers to technology use during the COVID-19 pandemic for PWMCs^a^ and their CGs^b^.

Theme	Description	Exemplary quotes
Limitations of existing technology	CGs and PWMCs experienced various frustrations and difficulties using technology during or prior to the pandemic due to technological limitations.	“Sometimes, you have a hard time hearing on Zoom . . . and it’s harder to focus. So you have to really focus on the screen.” [Louis, M^c^, 47 years, CG] “Sometimes, he does text . . . he does have parkinsonism, where he does now have more motor issues, [and] it’s hard for him to tap on the phone.” [April, F^d^, 62 years, CG] “People with memory loss can’t [usually] use a smartphone, and so, they’ve had to go to, like, a flip phone just because it keeps it simple . . . I get confused once in a while on the apps, but not too often.” [Lee, M, 77 years, PWMC]
Technological literacy	CGs and PWMCs varied in how familiar they were using technology.	“I have a newer cell phone, and it does more than what I can do . . . I’m not using everything that’s available there.” [Lily, F, 64 years, CG] “Lief was really very familiar with technology early on . . . he’s significantly lost his ability to navigate, with how rapidly changing technology has been.” [April, F, 62 years, CG] “I don't like apps and the security aspect of apps. I don't trust it . . . because I don't really understand it.” [Judy, F, 62 years, CG]
Dependence on CGs to use technology	PWMCs varied in their level of dependence upon their CGs to use technology.	“He is coming to me to help him with [the smart TV], which is okay. He's not as frustrated with asking for help as he used to be, but that's a thing that sometimes creates anxiety.” [Lily, F, 64 years, CG] “Charles sometimes gets frustrated at the new technologies. I have to keep helping him with it.” [Annmarie, F, 73 years, CG] “I might have to have instructions, but if I use it enough, it’ll go.” [Charles, M, 77 years, PWMC]

^a^PWMC: person with memory concerns.

^b^CG: caregiver.

^c^F: female.

^d^M: male.

#### Limitations of Existing Technology

Most technologies were not designed to suit the needs and abilities of PWMCs. Challenges for PWMCs using CMC included the absence of nonverbal cues, system lag-time issues, and difficulty learning, which resulted in less satisfaction in social interactions while using the technology. CMC can cause PWMCs to become confused or frustrated due to the lack of nonverbal cues and lag-time issues. Peggie (F, 60 years, CG) explained that

Not being able to see the body language, and engaging people appropriately, I think, is harder . . . for someone like [my husband], who maybe is having some challenges getting the words out . . . on a video it's harder.

Although some technologies were designed for PWMCs, a few CGs expressed that they were still too complicated and caused stress and confusion. Peggie shared that her husband’s memory loss support group was over the phone because the group members were unsuccessful in joining the videoconference due to difficulty learning new technology. Dementia severity impacted the ability to learn new technologies and limited the types of technologies that were useful to PWMCs.

#### Technological Literacy

CGs and PWMCs in the sample varied in their familiarity and comfort with technology; some participants felt well versed or tried to stay up to date on new technologies, while others tried to avoid using any and did not stay up to date. Rick (M, 73 years, CG) explained that

Layla and I use computers every day. We have iPhones, iPad, 2 laptops, Apple, [and] an iMac. We have Amazon echoes. We have cameras and security devices. Our thermostat . . . we do all that stuff.

Conversely, Gary (M, 77 years, CG) had a nonsmartphone cellular device, and while he used the computer regularly, the interview was his first video call. One PWMC regularly wrote blog posts and used CMC, such as social media with friends, while Myles (M, 85 years, PWMC) said,

I’m what is known as computer illiterate, so I have a difficult time using the telephone.

Lack of familiarity with older technology frequently precluded adoption of new technology.

Most CGs expressed interest in using new technology to benefit themselves and their relatives yet were often too intimidated to try. Peyton (F, 37 years, CG) shared,

I'm not really good with it . . . I want to stay up to date, but I don’t know what I'm doing.

Less technologically literate participants were frequently intimidated by various aspects of technology. Some participants chose not to use any technology that required more than passive use, such as downloading an app. The level of technological literacy prior to the pandemic impacted how likely a participant was to adopt a new technology during the pandemic.

Some CGs who were less technologically literate were able to seek out and try new technology with the help from others within their social network. April’s (F, 62 years, CG) uptake of new technologies was driven by her children:

I wouldn’t even consider it if my kids didn’t say “Hey, look at how cool this works . . . you should get this, Mom.”

Another CG, Barb, (F, 86 years) shared how her granddaughter often helped her learn a new technology, such as Zoom. Less technologically literate participants whose social networks reinforced the use of new technology were able to realize the benefits it had in their lives.

#### Dependence on Caregivers to Use Technology

Over half of the dyads shared various ways in which the PWMC was dependent on the CG to utilize technology. Annmarie (F, 73 years, CG) would completely set up Zoom so the PWMC could use it, while other PWMCs used technology independently and relied on their CGs only when problems arose, such as a screen going blank. Some CGs like Peggie (F, 60 years, CG) expressed a desire for the PWMCs to be more independent using technology:

I would really love it if he didn’t have to say, “Peggie, can you come help me make this happen?”

To maintain a technology’s usefulness, a CG had to be readily available, have time to teach the PWMC how to use it, and reinforce its use.

#### Overcoming Challenges

Participants who were motivated to try a new technology and overcame the associated challenges reported increased social connection, reduced the caregiving burden, and, in some instances, increased PWMC independence. Some CGs began using technology to promote learning and cognitive engagement among their relatives during the pandemic. For example, Zoom meetings and viewing pictures on Facebook helped to stimulate memory and mental capabilities to maintain recognition and memories of friends and family. Throughout the interviews, there were examples of PWMCs learning a new technology and gaining independence. Lisa (F, 77 years, CG) explained how she used to set up calls on her husband’s hearing phone so that he could call other people:

But since COVID he started using it himself . . . I said [to Myles], “That’s a little bit of freedom that you’ve got back.”

These fragments of increased PWMC freedom were able to slightly reduce caregiving burden despite increased stress and isolation.

## Discussion

### Principal Findings

The COVID-19 pandemic forced a shift in technology adoption for those who were fortunate enough to have access to it. Yet, CGs and PWMCs were not always willing to try new technology to assist them due to barriers such as each person’s level of technological literacy, the dependence on the CG for use, and limitations of the technology. For CGs and PWMCs who adopted new technology or adapted existing technology, technology was perceived as more useful during the pandemic than before, specifically in relieving boredom, maintaining social connections, and increasing CG respite.

### Comparison With Prior Work

Our research affirms that social network support, perceived value, perceived impact on the quality of life, and confidence in the ability to learn a new technology are all important influences of technology adoption [14,15]. Each of these influences played a role in technology adoption among PWMCs and CGs during the pandemic. Our research also highlights the role of technology in reducing the caregiving burden during the pandemic, along with increasing PWMC independence. GPS technology seemed to minimize CG stress by reducing the likelihood of PWMCs getting lost [23]. Similar to findings by Øderud et al [24], our research also shows that GPS technology provides CGs with respite time, while allowing PWMCs to enjoy their freedom and outdoor activities safely.

Studies suggest that assistive technology can reduce the caregiving burden yet may also pose an additional burden when technology adoption and use require too much CG help [25]. Such results are consistent with our findings that CGs are unlikely to adopt burdensome technology. In a review of 56 studies, assistive technology was perceived as removing CG stress and burden overall, although no significant change in the caregiving burden was reported in any of the 16 (29%) quantitative studies included in the review [23]. This discrepancy in CG burden results may be due to insensitivity of existing quantitative outcome measures [26]. Perhaps alternatively, no single device or app is comprehensive enough to reduce the caregiving burden, and instead, a combination of technologies is required to significantly reduce burden.

Technology can foster social connections for CGs and PWMCs by counteracting the impact of diminished social support and interactions during the pandemic. A systematic review of 25 publications concluded that information and communication technologies (ICT) can be an effective way of reducing social isolation among older adults; however, it is not suitable for all older adults [12]. Prior to the pandemic, technological interventions to reduce social isolation were understudied [27]. Not only are more ICT interventions being developed to reduce loneliness and increase social participation during the pandemic, but older adults also have a more positive view of ICT interventions now in contrast to before the pandemic [28]. Preliminary findings are showing promising results; in a qualitative study, researchers demonstrated that virtual memory cafés, where PWMCs and CGs remotely interact with other PWMCs and CGs, were able to support the social connectedness of PWMCs and CGs during the COVID-19 pandemic [29].

### Recommendations

The findings emphasize the importance of incorporating technological barriers of PWMCs and their CGs into the design process. Although dependence on the CG to use technology cannot be eliminated entirely, it should be avoided or minimized through mindful design. Therefore, it is important to engage both the PWMC and the family CG during each stage of the design process. Additionally, it is likely not feasible for technology developers to improve a users’ technological literacy. However, testing new products by CGs and PWMCs at various stages of dementia progression could highlight difficulties for less technologically literate users. This codesign process is important for any technology used by older adults yet is most important for technology designed specifically for PWMCs.

### Limitations

Limitations of this research include factors that may make the study population different from the general population. Participants were recruited through email, and they had to be willing and able to participate in interviews over Zoom, which could have led to selection bias. Participants may be more likely to use Zoom and other technologies than individuals who would have only been recruited in a nontechnological manner or did not have the capability to participate in a Zoom interview. Since income, education, and race/ethnicity are major influences of technology adoption, it is important to note that this sample was highly educated, had a median income higher than the national median income, and primarily identified as non-Hispanic White [30]. Additionally, we did not include PWMCs living in long-term care, and no PWMCs were in the later stages of dementia. Due to these differences, participants in this study may have different patterns of technology use/disuse compared to other CGs and PWMCs, all of which likely limit the generalizability of the findings. The results are hypothesis generating, and future research should engage a more racially and ethnically diverse population of CGs and PWMCs and include participants with lower incomes, educational attainment, and technological literacy.

### Conclusion

This research contributes to the literature on this population’s technology usage. Much of the existing literature focuses solely on assistive technology, while our research points out that mainstream technologies, such as smartphones or Zoom, were predominately used by this sample. Further research is needed to examine how mainstream technologies are used to support PWMCs and CGs in their everyday lives and to compare whether those who adopted more technology during the pandemic coped better with isolation than those who did not alter their technology usage.

Our research found that the COVID-19 pandemic resulted in an increase in technology use among many participants. These findings emphasize the importance of technology use among CGs and PWMCs, particularly during isolation, to provide relief from caregiving burden and afford PWMCs more independence.

## Appendix

Multimedia Appendix 1 Semistructured interview questions.

## Abbreviations

AD: Alzheimer disease
ADRD: Alzheimer disease–related dementias
CG: caregiver
CMC: computer-mediated communication
F: female
GPS: Global Positioning System
ICT: information and communication technologies
M: male
PWMC: person with memory concerns
UBACC: University of California, San Diego Brief Assessment of Capacity to Consent
