# Supplementary material for: The Use of Technology Among Persons With Memory Concerns and Their Caregivers in the United States During the COVID-19 Pandemic: Qualitative Study
Source: JMIR Aging. 2022 Mar 17;5(1):e31552. doi: 10.2196/31552 (PMC8972107; doi:10.2196/31552)
Supplement: Multimedia Appendix 1 [file aging_v5i1e31552_app1.pdf]

Appendix 1. Semi-structured interview questions

1. Before the COVID-19 pandemic, how did you typically use technology to engage with each other (i.e., caregiver/recipient dyad), your family, and friends? This might be things like emails, messaging, video calls, and social media.

2. Before the COVID-19 pandemic, how did you typically use technology to purchase necessities, such as medications, food, and toiletries?

3. Before the COVID-19 pandemic, how did you typically use technology to assist in caregiving, medical needs and health care? Did you engage with any of your medical providers online, such as electronic medical records and telehealth visits?

4. Has your use of technology changed in response to the COVID-19 pandemic?
    - Probe for *social engagement*, such as video calls, messaging, and social media; *employment*, such as working from home; *online shopping*; *caregiving*; *telehealth/medical care; online finances/banking*].

5. Has technology helped you cope with the COVID-19 pandemic (e.g., to diminish boredom, stay connected to others)?

6. What challenges and frustrations have you encountered during the COVID-19 pandemic when using technology to engage with each other, your friends and family, and medical providers?
    - Probe for more specifics on: social engagement? care provision? etc.

7. What are the benefits to using technology during and after the COVID-19 pandemic to engage with each other, your friends and family, and medical providers?
    - Probe for more specifics on: social engagement? care provision? etc.
